# Workplace gender composition and sickness absence: A register-based study from Sweden

**DOI:** 10.1177/14034948231176108

**Published:** 2023-06-02

**Authors:** Inger Haukenes, Anne Hammarström

**Affiliations:** 1 Department of Global Public Health and Primary Care, University of Bergen, Norway; 2 Research Unit for General Practice, NORCE – Norwegian Research Centre, Norway; 3 IMM, Karolinska Institutet, Sweden; 4 Department of Epidemiology and Global Health, Umeå University, Sweden

**Keywords:** Sick leave, sick days, absenteeism, gender, workplace, labour market, gender segregated workplaces, gender composition, registry, Sweden

## Abstract

**Aims:**

This study aimed to examine the association between gender composition in the workplace and sickness absence days during a one-year period.

**Methods:**

The study population was drawn from the Northern Swedish Cohort (wave 3; 2007) by Statistics Sweden and consisted of all participants belonging to a specific workplace (*n*=837) as well as all co-workers at the workplace of the participants (*n*=132,464; 67,839 women and 64,625 men). Exposure was the gender composition of the workplace, and outcome was cumulative sickness absence days (*⩾*90 days or not) during 2007, provided through a link to the Database for Health Insurance and Labour Marked Studies of Statistics Sweden. Covariates were gender, age, educational level and branch of industry from the same data source. We performed descriptive analyses and multivariable regression analyses.

**Results:**

Workers in extremely female-dominated workplaces had a significantly higher risk of cumulative sickness absence days (⩾90 days) compared with gender-equal workplaces (fully adjusted odds ratio (OR)=1.27; 95% confidence interval (CI) 1.09–1.48), whereas those working in extremely and moderately male-dominated workplaces had a significantly lower sickness absence risk (OR=0.62 and 0.66, respectively). Stratified by gender, the higher absence risk at female-dominated workplaces was fully explained by variation in branches of industry. Women working in extremely male-dominated workplaces had a significantly lower absence risk (OR=0.75), as did men working in moderately male-dominated workplaces (OR=0.78).

**Conclusions:**

**Workplaces dominated by women had a significantly higher risk of days lost to sickness absence compared to gender-equal workplaces. Stratified by gender, this higher risk was explained by branch of industry.**

## Introduction

The Scandinavian countries are characterised by high awareness of gender equity in policy and practice, as well as high employment rates among men and women. The Nordic welfare model includes the public care of children and the elderly – a sector of employment that to a large extent is occupied by women. Despite significant progress in gender equality over the last decades, there is still relatively strong gender segregation in the Scandinavian labour market [1]. In addition, there is a consistently higher rate of sick leave among women compared with men [2]. The question at stake in this study is to what extent gender composition in the workplace impacts the likelihood of cumulative days lost to sickness absence.

In general, the nature of the work in the public sector may be characterised as ‘caring for others’, whereas work in the private sector is more often related to construction, production and maintenance of physical objects. Thus, horizontal gender segregation situates men and women in different occupations and workplaces, whereas vertical gender segregation situates men and women in different positions of power and control over work [3]. The gendered structure of the labour market is historically defined by the gender order in society, in which men’s domination over women is created and maintained [4].

To date, studies have found a U-shaped pattern in the association between gender composition in the workplace and sickness absence, meaning that workplaces with equal gender distribution tend to have the lowest sickness absence, and workplaces dominated by one gender have the highest [5–10]. However, the strength and consistency of the U shape seem to vary with the organisational level taken as exposure (branch, occupation and workplace), the outcome measures (sickness absence days, spells and length of spells) [10] or whether the U shape reflects crude or adjusted risk [5–9]. There is a shortage of studies using the sum of sickness absence days as outcome, although this might be particularly relevant in the context of public health and sustainable work participation.

In most studies of sick leave using measures of gender composition as exposure, there seems to be an agreement that occupation, branch of industry or occupational social class are the most relevant confounders [5,7–9]. However, there is strong evidence that education as an upstream determinant of health is related to sickness absence [11,12]. Thus, there is a need for large registry studies taking account of both education and occupational data in the association between gender-segregated working life and sickness absence.

With respect to theories of how gender composition drives the sickness-absence pattern in workplaces, there are three main views. The first view addresses the adverse effects of being the gender in minority, caused by mechanisms such as assimilation, stereotyping and polarisation [13]. The second thematises absence culture or norms that develop in workplaces dominated by one gender [9,14]. The third view considers that exposure to work-related risk factors in workplaces dominated by men or women contribute an absence profile consistent with the U shape [15,16]. Typically, these theories are used to explain the U-shaped pattern of sickness absence in the gendered labour market. However, there are still doubts about the strength and consistency of the U shape, and therefore large register studies are warranted.

### Aims

The aim of this study was to examine the association between gender composition in the workplace and working days lost to sickness absence (⩾90 days) during a one-year period in a large data set based on a representative sample of the Swedish population.

## Methods

### Population

The study population was based on the Northern Swedish Cohort, consisting of all pupils in Luleå in 1981 attending their last year of compulsory school (age 16; *n*=1083, 506 girls and 577 boys). The cohort was followed up with questionnaires at ages 16, 18, 30 and 42 years. In 2007 (participants’ age=42 years), the response rate was 94.3% (*n*=1010) of those alive (*n*=1071). Socio-demographic characteristics, socio-economic status and health status were representative of the Swedish population [17].

In 2007, Statistics Sweden identified all participants in the Northern Swedish Cohort who belonged to a specific workplace (*n*=837), excluding those who did not work or lacked a street address for the work site (*n*=173). Second, all co-workers at workplaces of the participants in the cohort were identified and added to the total study sample, giving a total of 135,398 potential participants (69,194 women and 66,204 men). From these data, the gender composition of the workplace, in which all employees work at the same site (street address), was calculated. The labour market structure of Luleå was comparable to Sweden as a whole regarding the distribution of branches of business in 2007 [18]. To avoid including small family businesses, with frequent part-time positions and gender compositions shaped by family conditions, employees working at workplaces with fewer than 10 co-workers were excluded (*n*=645; 0.5% of the population). Participants outside the age range of the regular workforce in Sweden (<15 and >64 years were excluded; *n*=1863). Participants with missing data on educational level were also excluded (*n*=426). The final study population consisted of 132,464 individuals (67,839 women and 64,625 men).

### Data collection

Register data (gender, age, educational level, branch of industry and cumulative sickness absence days) were collected from the Longitudinal Integration Database for Health Insurance and Labour Marked Studies of Statistics Sweden (2007) and linked to the Northern Swedish Cohort using the personal identification number given to each Swedish citizen.

### Outcome

The outcome was cumulative days of certified sickness absence during 2007, thus reflecting the sum of days of shorter and longer absence spells. Sickness absence spells that were shorter than 16 days are not recorded in the registry. Absence is measured as net sickness absence days; that is, 10 days with 100% sickness absence is counted as 10 days, whereas 10 days with 50% sickness absence is counted as five days. Net sickness absence days were recoded to a dichotomous variable (<90 days=0 and ⩾90 days=1).

### Exposure

Gender distribution in the workplace was coded according to five categories, with percentages related to female workers in the workplace (⩽20%, 21%–40%, 41%–60%, 61%–80% and >80%).

### Covariates

Education (International Standard Classification) was registered in seven categories and recoded into six categories: university >3 years, post upper secondary ⩾3 years, post upper secondary <3 years, upper secondary 3 years, upper secondary <3 years and pre upper secondary. Age was recoded in categories commonly used by Statistics Sweden (SCB) and the EU (15–24, 25–34, 35–44, 45–54, 55–64 years) [19]. The Swedish Standard Industrial Classification 2002 (SNI 2002) was recoded into 13 main branches according to the Standard. The branches ‘agriculture’ and ‘fishing’ were merged with ‘mining’ due to low numbers.

### Analysis

Descriptive statistics present the distribution (*n*, %) across age groups, educational levels, gender composition in the workplace and branches of industry among men and women separately, along with the proportion (%) reaching ⩾90 days of accumulated sickness absence during 2007. In addition, we present results from univariabel logistic regression analyses examining the association between exposure and covariates, and the outcome. Reference groups for age, education and gender composition were those presumed to have the lowest risk for sickness absence ⩾90 days, that is, the youngest age group, the highest educated and gender-equal workplaces. For branches of industry, ‘financial intermediation’ was chosen due to the equal gender distribution.

We performed three logistic regression analyses introducing age, educational level and branch of industry in three models of adjustments. First, we examined the association between gender composition in the workplace and accumulated sickness absence ⩾90 days in the total population and thereafter similar analyses for men and women separately. To examine the robustness of our findings, we performed sensitivity analyses, with cut-offs for cumulative sickness absence days at ⩾30 days and ⩾60 days.

Results are presented as odds ratios (ORs) with 95% confidence intervals (CIs). The analyses were performed by use of PASW Statistics for Windows v18 (SPSS, Inc., Chicago, IL).

### Ethical approval

The regional ethics vetting board in Umeå, Sweden, approved the study protocol.

## Results

More women (43.4%) than men (34.2%) worked in extremely gender-segregated workplaces with dominance of their own gender, whereas more men (31.2%) than women (22.0%) worked in slightly less gender-segregated workplaces with dominance of their own gender. ‘Manufacturing’ occupied 36.4% of the men, whereas ‘human health and social work’ occupied 54.9% of the women. Women had more than twice the prevalence of one-year cumulative sickness absence days (⩾90 days) than men (4.5% for women vs. 1.8% for men). Sickness absence (⩾90 days) increased by higher age and lower education. With respect to branch of industry, ‘transportation, storage and communication’ had the highest prevalence of sickness absence days (2.9% among men and 5.5% among women; Table I).

**Table I.** Distribution of men and women and associations with one-year cumulative sickness absence ⩾90 days across age groups, education, gender composition in the workplace and branch of industry.

	Men					Women				
	Sickness absence ⩾90 days					Sickness absence ⩾90 days				
	*n*	(%)	%	OR	95% CI	*n*	(%)	%	OR	95% CI
Gender	64,625	–	1.8			67,839	–	4.5		
Age groups (years)										
15–35	17,993	27.8	0.8	1		17,861	26.6	2.5	1	
36–45	19,282	29.8	1.5	1.78	1.46–2.18	19,460	28.7	4.3	1.75	1.56–1.97
46–55	15,981	24.7	2.1	2.60	2.14–3.16	17,773	26.2	5.2	2.12	1.89–2.37
56–64	11,369	17.6	3.7	4.69	4.69–5.67	12,745	18.8	6.7	2.78	2.48–3.13
Highest educational level										
Master or doctoral	3324	5.1	1.3	1		1804	2.7	2.5	1	
Bachelor or equivalent ⩾3 years	20,423	31.6	1.1	0.87	0.63–1.21	26,685	39.3	3.3	1.30	0.96–1.75
Post upper secondary 2 years	11,865	18.4	1.4	1.12	0.80–1.58	12,448	18.3	4.5	1.79	1.32–2.43
Upper secondary 3 years	12,262	19.0	1.8	1.40	1.01–1.95	10,428	15.4	4.4	1.76	1.30–2.40
Upper secondary 2 years	12,671	19.6	3.1	2.50	1.82–3.45	13,710	20.2	6.7	2.76	2.05–3.73
Primary	4080	6.3	3.6	2.94	2.08–4.16	2764	4.1	7.2	2.98	2.15–4.13
**Gender composition at the workplace** %=women										
0–20%: extremely male dominated	22,080	34.2	1.9	1.06	0.89–1.25	3234	4.8	3.2	0.90	0.72–1.12
21%–40%: male dominated	20,184	31.2	1.6	0.91	0.76–1.09	8258	12.2	3.5	0.97	0.83–1.13
41%–60%: gender equal	11,454	17.7	1.8	1		11,981	17.7	3.6	1	
61%–80%: female dominated	5189	8.0	2.2	1.26	1.00–1.59	14,931	22.0	4.5	1.27	1.13–1.44
80%–100%: extremely female dominated	5718	8.8	2.2	1.26	1.01–1.58	29,435	43.4	5.3	1.52	1.37–1.70
**Branches of industry (SNI 2002)**										
1. Agriculture, fishing, mining (A/B)	1608	2.5	1.8	1.55	0.96–2.46	254	0.4	2.0	0.80	0.32–1.99
2. Manufacturing (D)	23,539	36.4	1.8	1.53	1.13–2.09	6185	9.1	3.6	1.50	1.18–1.91
3. Electricity, gas and water supply (E)	384	0.6	1.0	0.88	0.32–2.46	89	0.1	4.5	1.88	0.68–5.23
4. Construction (F)	1429	2.2	2.3	1.98	1.26–3.21	209	0.3	3.8	1.59	0.76–3.32
5. Wholesale and retail trade, repair motor vehicles (G)	1348	2.1	2.4	2.03	1.29–3.21	1175	1.7	3.3	1.37	0.94–2.00
6. Hotels and restaurants (H)	342	0.5	1.5	1.24	0.49–3.15	489	0.7	4.9	2.06	1.31–3.26
7. Transportation, storage and communication (I)	4825	7.5	2.9	2.54	1.81–3.55	3244	4.8	5.5	2.33	1.82–3.00
8. Financial intermediation (J)	3807	5.9	1.2	1		3973	5.9	2.4	1	
9. Real estate, renting and business (K)	4862	7.5	1.7	1.45	1.01–2.09	2739	4.0	3.8	1.59	1.20–2.11
10. Public administration and defence (L)	8797	13.6	1.1	0.97	0.68–1.38	5610	8.3	3.2	1.31	1.02–1.68
11. Education (M)	3774	5.8	2.0	1.70	1.17–2.46	4980	7.3	4.3	1.79	1.34–2.28
12. Human health and social work (N)	8237	12.7	2.2	1.90	1.37–2.64	37221	54.9	5.2	2.19	1.78–2.69
13. Other community, social and personal service (O)	1669	2.6	2.2	1.84	1.18–2.87	1663	2.5	3.1	1.29	0.92–1.82

Higher age was statistically significant associated with sickness absence days (⩾90 days) compared with those of youngest age. Among women ORs ranged from 1.75 among workers who were 36–45 years of age to 2.78 among workers who were 56–64 years of age. We found an educational gradient in risk of sickness absence among women, with ORs ranging from 1.30 among workers with a bachelor’s degree or equivalent education to 2.98 among workers with primary education only compared with the highest educated. Workers within ‘transportation, storage and communication’ had the highest risk of sickness absence (OR=2.54 among men and OR=2.33 among women), followed by ‘wholesale and retail trade, repair motor vehicles’ among men (OR=2.03) and ‘human health and social work’ among women (OR=2.19), all compared with their respective reference group ‘financial intermediation’ (Table I).

In the total study population, the odds of one-year cumulative sickness absence (⩾90 days) were significantly higher among workers in extremely female-dominated workplaces (fully adjusted OR=1.27 (95% CI 1.09–1.48), and significantly lower in extremely and moderately male-dominated workplaces (OR=0.62 and 0.66, respectively) compared to gender equal workplaces. Adjustment for branches of industry attenuated the ORs among female-dominated workplaces substantially (Table II).

**Table II.** Age-adjusted odds ratios and 95% confidence intervals for one-year cumulative sickness absence ⩾90 days among men and women by gender composition in the workplace adjusted for age, education and branch of industry.

Gender composition	Crude		+Age		+Education		+Branch of industry	
	OR	95% CI	OR	95% CI	OR	95% CI	OR	95% CI
0–20%: extremely male dominated	0.76	0.67–0.85	0.82	0.73–0.93	0.68	0.60–0.76	0.62	0.53–0.73
21%–40%: male dominated	0.80	0.71–0.89	0.82	0.74–0.92	0.77	0.68–0.86	0.66	0.57–0.77
41%–60%: gender equal	1		1		1			
61%–80%: female dominated	1.47	1.32–1.64	1.45	1.30–1.61	1.40	1.26–1.56	1.08	0.93–1.25
80%–100%: extremely female dominated	1.83	1.67–2.01	1.83	1.67–2.01	1.82	1.66–2.00	1.27	1.09–1.48

Among men only, the final adjustment for branches of industry attenuated the OR in extremely female-dominated workplaces from 1.43 (95% CI 1.14–1.79) to an insignificant level, whereas the OR in moderately male-dominated workplaces became significant (OR=0.78, 95% CI 0.61–0.99; Table III). Among women only, adjusting for branches of industry attenuated the ORs in extremely female-dominated workplaces (OR=1.52, 95% CI=1.36–1.70) and moderately female-dominated workplaces (OR=1.23, 95% CI 1.09–1.40) to insignificant levels, whereas the OR in extremely male-dominated workplaces became significant (OR=0.75, 95% CI 0.58–0.98; Table IV). All estimates are compared with the gender-equal reference group.

**Table III.** Age-adjusted odds ratios and 95% confidence intervals among men only for one-year cumulative sickness absence ⩾90 days by gender composition at the workplace, adjusted for age, education and branch of industry.

Gender composition	Crude		+Age		+Education		+Branch of industry	
	OR	95% CI	OR	95% CI	OR	95% CI	OR	95% CI
0–20%: extremely male dominated	1.06	0.89–1.25	1.20	1.01–1.42	0.88	0.74–1.05	0.87	0.68–1.11
21%–40%: male dominated	0.91	0.76–1.09	0.95	0.79–1.13	0.85	0.71–1.02	0.78	0.61–0.99
41%–60%: gender equal	1		1		1		1	
61%–80%: female dominated	1.26	1.00–1.59	1.22	0.97–1.55	1.22	0.97–1.54	1.06	0.78–1.44
80%–100%: extremely female dominated	1.26	1.01–1.58	1.29	1.03–1.06	1.43	1.14–1.80	1.15	0.79–1.68

**Table IV.** Odds ratios and 95% confidence intervals among women only for one-year cumulative sickness absence ⩾90 days by gender composition at the workplace, adjusted for age, education and branch of industry.

Gender composition	Crude		+Age		+Education		+Branch of industry	
	OR	95% CI	OR	95% CI	OR	95% CI	OR	95% CI
0–20%: extremely male dominated	0.90	0.72–1.12	0.99	0.80–1.23	0.88	0.71–1.10	0.75	0.58–0.98
21%–40%: male dominated	0.97	0.83–1.13	1.03	0.88–1.20	0.97	0.83–1.13	0.79	0.65–1.96
41%–60%: gender equal	1		1		1		1	
61%–80%: female dominated	1.27	1.13–1.44	1.25	1.10–1.41	1.23	1.09–1.40	0.96	0.81–1.13
80%–100%: extremely female dominated	1.52	1.37–1.70	1.51	1.35–1.69	1.52	1.36–1.70	1.09	0.91–1.30

Sensitivity analyses with cut-offs for cumulative sickness absence at ⩾30 days and ⩾60 days showed a similar trend (see Supplemental Material for the total study population).

## Discussion

### Main findings

In a comprehensive register study from Sweden, we examined the relation between gender composition in the workplace and working days lost to sickness absence (⩾90 days) during a one-year period, taking account of age, education and branches of industry. We found that men and women working in extremely female-dominated workplaces had a significantly higher likelihood of working days lost to sickness absence (⩾90 days), whereas those working in male-dominated workplaces had a significantly lower absence risk, all compared with gender-equal workplaces. Adjusting for branches of industry had a substantial impact on the relation. Among men and women separately, the higher absence risk in female-dominated workplaces was explained by branches of industry.

### On the issue of measuring sickness absence

As early as in 1998, Hensing et al. warned about the large variety of sickness absence measures used in studies [20] and the lack of grounds for choosing one or the other. It is generally accepted to use short-term and long-term sickness absence. However, there is no consensus on cut-offs for days. In working life, higher numbers of days lost to sickness absence are likely to challenge productivity and impose work stress among those present at work. Thus, we decided to use the cumulative number of days lost to sickness absence for one year and set the cut-off at ⩾90 days in order to examine if a high burden of sickness absence was related to gender composition in the workplace.

### The sickness absence pattern

The current study did not support the U-shaped pattern of sickness absence characterised by higher absence in workplaces where one gender is in the majority relative to workplaces with a more equal gender distribution. Rather, we found a significantly lower likelihood of days lost to sickness absence in male-dominated workplaces and a significantly higher likelihood among female-dominated workplaces. Our findings are partly in line with a comprehensive register study from Sweden concluding that gender composition in occupations and branches plays a significant role in sickness absence among men and women, particularly for those working in extremely female-dominated occupations [6]. Moreover, this previous study argues that work-related hazards in occupations/branches seem to influence sickness absence more than the gender composition as such. In the present study, we found a limited effect from adjusting for age and education, whereas branches of industry (SNI 2002), reflecting the type of work performed at workplaces, had substantial explanatory value. However, where branches of industry explained large parts of the absence risk at female-dominated workplaces, the risk among male-dominated workplaces became negative compared with the gender-equal reference group. Among the differences between the Swedish register study and the present study is the outcome measure. Lidwall used the first sick-leave spell >14 days [6], whereas the current study used cumulative sickness absence ⩾90 days.

One explanation could be that women are sick listed with shorter spells more often than men [21], thus accumulating ⩾90 days more easily. In addition, men are more reluctant to seek health care, thus avoiding shorter spells of sickness but ultimately ending with a longer sickness absence spell (>14 days) when eventually seeking help. Thus, different sick-leave measures may yield different results and thereby add valuable knowledge to the research field.

A group of studies found that an increased proportion of women at the workplace drives the sickness absence among women only, and vice versa for men [8,9,22]. Mastekaasa, taking account of both workplace gender composition and occupation, found that men’s sickness absence (>14 days) was relatively unrelated to gender composition, whereas a relation among women working at female-dominated workplaces was present but weak [9]. Bryngelson et al.’s result was somewhat similar, although they found a stronger impact among women working in female-dominated workplaces on their first episode of long-term sickness absence [8]. Finally, Laaksonen found that men and women working at female-dominated workplaces and occupations had a higher risk of short-term sickness absence but not intermediate or long-term absence [22]. Summing up, men’s sickness absence seems to be less influenced by gender composition than women’s absence does. Moreover, burdens or hazards in occupations probably play a more important role than gender composition as such. Finally, the sickness-absence measures applied must be carefully considered when interpreting the findings.

### Explanations of the gendered pattern

Female-dominated occupations often imply a degree of emotions, that is, the ‘right’ emotions to care for customers, clients, patients and pupils in a respectful manner. A review of health effects associated with emotional labour found that workers who felt the need to manage and regulate their emotions at work were more likely to develop symptoms of burnout, anxiety and depression compared to those who more easily expressed the ‘right’ emotions [23]. Stressful periods in life may also challenge the capacity to express the ‘right’ emotions at work and push the worker closer to an absence period caused by stress-related illness.

Moreover, work in female-dominated occupations in the public sector are characterised by shift work, part-time positions (voluntary or involuntary) and limited control and flexibility in the work situation. The literature finds an increased risk of poor health and sickness absence among those working in environments that hold these characteristics [24], particularly exposure to low control and high strain [25]. Finally, a study among young people in Sweden showed that the recession of the 1990s led to gendered health consequences; women developed poorer health than men due to cutbacks in female-dominated occupations and subsequent lack of control and increased demands [26]. Summing up, the nature of the work in female-dominated occupations often requires caring emotions and respectful conduct to achieve the aims of the job. This work also tends to hold less job control, higher job strain, more part-time positions and lower income than male-dominated work. In the current study, branches of industry reflecting the type of work performed at workplaces explained a large part of the sickness-absence risk related to female domination in the workplace and fully explained the risk in gender-stratified analyses. As most women, but also an increasing number of men, work in the public sector within education, health and social work, it is reasonable to infer that these branches hold risk factors for accumulating sickness-absence days more easily than branches dominated by men.

### Strengths and limitations of the study

Strengths of the current study include the population’s size and the register-based data being complete and reliable, with no loss to follow-up. One limitation is that the registry records sick leave from day 16 onwards, not the first 15 days which are paid by the employer. Hence, it is likely that several participants reached 90 days without being included in the exposed group. However, selection into the exposed group (⩾90 days of sick leave) versus the control group (<90 days) is proportional and not differential and will not bias the results. Second, we were not able to adjust for important work-related factors, such as demands and control. However, adjusting for branch of industry has the potential to take account of important work-related factors. Third, the cross-sectional design makes it impossible to draw any conclusions about causality. Finally, the population has a similar age distribution as the general population in Sweden but with a somewhat higher level of education. The generalisation of the results may be limited to countries with a gender-segregated labour market and high labour-market participation among women.

## Conclusions

Men and women in workplaces with extreme female domination had a significantly higher risk of days lost to sickness absence compared to gender-equal workplaces. Branches of industry explained a substantial part of the higher risk among men and women working at female-dominated workplaces. Future research should discuss the use of sickness-absence measures and modifiable risks more thoroughly.

## Supplemental Material

sj-docx-1-sjp-10.1177_14034948231176108 – Supplemental material for Workplace gender composition and sickness absence: A register-based study from Sweden Supplemental material, sj-docx-1-sjp-10.1177_14034948231176108 for Workplace gender composition and sickness absence: A register-based study from Sweden by Inger Haukenes and Anne Hammarström in Scandinavian Journal of Public Health
