# Supplementary material for: Workplace gender composition and sickness absence: A register-based study from Sweden
Source: Scand J Public Health. 2023 Jun 2;52(6):678–84. doi: 10.1177/14034948231176108 (PMC11308254; doi:10.1177/14034948231176108)
Supplement: sj-docx-1-sjp-10.1177_14034948231176108 – Supplemental material for Workplace gender composition and sickness absence: A register-based study from Sweden [file sj-docx-1-sjp-10.1177_14034948231176108.docx]

Supplemental Table. Age-adjusted Odds Ratios and 95% confidence intervals for one-year cumulative sickness absence ≥30 days and ≥60 days, respectively, among men and women by gender composition at the workplace, adjusted for age, education and branche of industry.

|  | ≥30 days of cumulative sickness absence | | | | | | | | | | |
| --- | --- | --- | --- | --- | --- | --- | --- | --- | --- | --- | --- |
|  | Crude | |  | + Age | |  | + Education | |  | + Branche of industry | |
|  | OR | 95% CI |  | OR | 95% CI |  | OR | 95% CI |  | OR | 95% CI |
| **Gender composition** |  |  |  |  |  |  |  |  |  |  |  |
| 0–20%: extreme men-dominated | 0.77 | 0.70–0.84 |  | 0.82 | 0.75–0.90 |  | 0.69 | 0.63–0.75 |  | 0.67 | 0.60–0.76 |
| 21%–40%: men-dominated | 0.78 | 0.72–0.85 |  | 0.80 | 0.74–0.87 |  | 0.75 | 0.69–0.81 |  | 0.70 | 0.63–0.78 |
| 41%–60%: gender equal | 1 |  |  | 1 |  |  | 1 |  |  | 1 |  |
| 61%–80%: women-dominated | 1.53 | 1.41–1.65 |  | 1.51 | 1.39–1.63 |  | 1.47 | 1.35–1.59 |  | 1.20 | 1.08–1.34 |
| 80%–100%: extreme women-dominated | 1.81 | 1.69–1.94 |  | 1.81 | 1.69–1.94 |  | 1.80 | 1.68–1.93 |  | 1.35 | 1.20–1.52 |
|  | ≥60 days of cumulative sickness absence | | | | | | | | | | |
| **Gender composition** |  |  |  |  |  |  |  |  |  |  |  |
| 0–20%: extreme men-dominated | 0.74 | 0.66–0.82 |  | 0.80 | 0.72–0.89 |  | 0.66 | 0.59–0.73 |  | 0.63 | 0.54–0.72 |
| 21%–40%: men-dominated | 0.79 | 0.72–0.87 |  | 0.81 | 0.74–0.90 |  | 0.76 | 0.69–0.84 |  | 0.68 | 0.60–0.77 |
| 41%–60%: gender equal | 1 |  |  | 1 |  |  | 1 |  |  | 1 |  |
| 61%–80%: women-dominated | 1.48 | 1.35–1.62 |  | 1.45 | 1.32–1.60 |  | 1.41 | 1.28–1.55 |  | 1.10 | 0.97–1.25 |
| 80%–100%: extreme women-dominated | 1.82 | 1.67–1,97 |  | 1.81 | 1.67–1,97 |  | 1.81 | 1.66–1,96 |  | 1.28 | 1.12–1,47 |
